# Supplementary material for: Epidemiology and Ecology of Usutu Virus Infection and Its Global Risk Distribution
Source: Viruses. 2024 Oct 12;16(10):1606. doi: 10.3390/v16101606 (PMC11512428; doi:10.3390/v16101606)
Supplement: Supplementary file 1 [file viruses-16-01606-s001.zip › viruses-3193768-supplementary.pdf]

**Appendix A**

**Additional Methods:**

**Assembling covariates**

We created a global grid-map with a resolution of 10 km × 10 km using ArcGIS 10.7 (Esri Inc, Redlands, CA, USA) and then associated each grid with ecological variables. The map in raster format was summarized into a processable data format at the study level based on the grid-map, and the R package "Raster" was used for resampling in order to change the resolution of raster data. Specifically, for land cover data, percentage coverage of 13 types were extracted and summarized at 10 km × 10 km resolution. For climatic data, 19 variables (Bio1–19) were created based on monthly maximum temperature, monthly minimum temperature, and monthly rainfall using the R package "dismo" in order to generate more biologically meaningful variables. For other variables, we used the "aggregate" function to create a new raster with a 10 km resolution, and then used the "projectRaster" function to project the values of environmental variables to the grid-map through a bilinear interpolation method. For a polygon occurrence record, we assigned the grid containing the centroid of the polygon as the occurrence grid. To minimize potential ecological fallacy, we first excluded all huge polygon occurrence records from ecological modelling due to insufficient resolution. For polygon occurrence records with an area no larger than 900 $km^2$, we calculated the mean of each ecological variable across all grids within the polygon and associated the mean value with the occurrence grids, that was, the grids containing the centroid of the polygon.

## Collecting occurrence data of vector and hosts

Vector

Considering that *Culex pipiens* is the most important vector for USUV, we conducted an exhaustive literature search and occurrence data search to identify relevant data. The databases included PubMed (https://pubmed.ncbi.nlm.nih.gov/), Web of Science (http://isiknowledge.com/), medRvix (https://www.medrxiv.org/), and bioRvix (https://www.biorxiv.org/). And the occurrence data was obtained from VectorMap (https://experience.arcgis.com/experience/5f95c3edfbea4634b8347fec0bd1dcd6/) and GBIF (https://www.gbif.org/). And *Aedes albopictus* and *Culiseta longiareolata* were another two important vectors of USUV, their occurrence data were mainly obtained from VectorMap and GBIF. Furthermore, the study conducted by Kraemer A et al. [47] also collected the distribution of *Aedes albopictus*, so we included them into our study. When processing GBIF source data, we excluded data from preserved specimen and fossil specimen.

In the process of searching literature on the occurrence of *Culex pipiens* in database, we excluded studies that met any of the following criteria: (I) not related to detection result of *Culex pipiens*; (II) testing insecticides, detection tools, drugs or vaccines; (III) focusing on molecular research of *Culex pipiens*; (IV) study regions beyond our research focus (Europe, Africa, and Middle East); (V) not provide point data; (VI) reviews and meta-analysis; (VII) not providing access to the full-text. In the end, 318 articles were included in our study (**Appendix 2)**.

Bird hosts

Before searching the occurrence data of main wildlife hosts, we first identified the three most important wildlife hosts through a series of steps:

a. Summarize the molecular and serological USUV positive proportion of various wildlife hosts.

b. In the summary data of molecular tests, the positive proportion of each species was sorted from high to low, and generated “Importance1” based on the order.

c. In the summary data of serological tests, the positive proportion of each species was sorted from high to low, and generated “Importance2” based on the order.

d. Due to approximately 72% of all positive data were from molecular test, we assigned weights of 72% and 28% to “Importance1” and “Importance2”, respectively, and generated “Weighted Importance”.

Weighted Importance = 0.72* Importance1+0.28* Importance2

Then, *Turdus merula*, *Passer domesticus*, and *Ardea cinerea* were selected as the main hosts. And the occurrence data of *Turdus merula*, *Passer domesticus*, and *Ardea cinerea* were obtained from GBIF, we also excluded data from preserved specimen, and fossil specimen.

**Modelling the vector and host environmental suitability**

Considering that the global allocation of scientific resources for species surveillance and research has been focused on rich, developed countries. It is thus likely that discovery is biased spatially. To deal with potential reporting bias due to imbalanced sampling efforts to collect and report data on *Culex pipiens*, *Aedes albopictus*, *Culiseta longiareolata*, *Turdus merula*, *Passer domesticus*, and *Ardea cinerea*, we

included the number of sequences available in GenBank as a proxy for study efforts, assuming that surveillance efforts for each species were proportional to the number of sequences available in GenBank [48]. The reciprocals of the normalized number of sequences in regions were used as weights of pseudo-absence locations sampling.

Climate, land cover, elevation, Leaf area index (LAI), and livestock density variables were chosen to explore the probability of three vectors and three bird hosts occurrences (**Table S4**). Before modeling, to reduce multicollinearity among selected variables, and to improve predictive accuracy of the models, we performed a variable selection based on their pairwise correlations using the R package "caret" as follows: (1) calculate the correlation matrix of all variables, (2) determine the two variables (variables A and B) with the largest absolute pairwise correlation coefficient, (3) determine the average correlation coefficients between A/B and the other variables, (4) include the variable A or B with a smaller average correlation coefficient in the analysis and exclude the other, (5) repeat steps 2–4 until no absolute correlation is above the threshold (in our study, 0.75) (Available at: https://cran.r-project.org/web/packages/caret/index.html).

Based on the distribution information of vectors and hosts species, *Culex pipiens, Aedes albopictus*, *Culiseta longiareolata*, *Ardea cinerea*, and *Passer domesticus* were distributed in Europe, Africa, and the Middle East, however, *Turdus merula* was only distributed areas north of 28°N latitude. Thus, during the modeling process, we also made corresponding adjustments to the modeling area.

We used Boosted Regression Trees (BRT) model (on a year scale from 2002 to

2023) to predict the probability of *Culex pipiens, Aedes albopictus*, *Culiseta longiareolata*, *Turdus merula*, *Passer domesticus*, and *Ardea cinerea* occurrence. When modeling, we randomly sampled pseudo missing locations within a range of 30-3000 kilometers around the location of the occurrence in ratios of 1:1, 1:3, and 1:5, respectively, while, due to the large number of positive data points of *Turdus merula*, *Passer domesticus*, and *Ardea cinerea*, we only conducted a 1:1 ratio sampling. We then fitted an initial model for each species, and predictors with relative contributions (RCs) greater than 0.5% were retained for the formal model building. In the final model, we randomly divided the data into an 75% training set and a 25% test set and fitted a BRT model, which was repeated 100 times [49-51]. That is, we obtained 100 models based on the 100 training datasets for each target species, to which we refer as a model assembly. Using these presence and pseudo-absence locations and ecological predictors, BRT models were fitted using the “gbm.step” function in “dismo” package in R 4.0.3 (R Foundation for Statistical Computing, Vienna, Austria) with a tree complexity of 4, a learning rate of 0.005, and a bagging fraction of 0.75 based on their satisfactory performance in our previous research [52,53]. A 10-fold cross validation was used to identify the optimal number of trees using the gbm.step function in the R package “dismo”. The Kappa coefficient and F1 Score for testing set and relative contributions of all selected predictors were averaged over the 100 models to represent the final predictive performance of the model and estimation results. The best threshold value used for final predictions of presence or absence of USUV was based on the Youden index derived from the average receiver operating characteristic

(ROC) over the 100 models [52]. By comparing Kappa coefficient and F1 Score of various models, we incorporate the probability of species occurrence predicted by the best model into the subsequent analysis.

**Predict the probability of USUV occurrence**

Random Forest

RF (Random Forest) is another classical ensemble learning model widely used [54]. The training algorithm of RF is based on bootstrap aggregating. Each tree is trained on many bootstrap samples, and was then evaluated using the remaining data to produce more accurate classifications. The unknown class of an observation will be calculated by the majority vote of the out-of-bag predictions for that observation [55]. We optimized the key learning parameter, mtry, which defines the number of variables randomly sampled as candidates at each split, between the range of 2–20 in random search, with a 10-fold cross-validation process to avoid overfitting. The R packages "caret" and "randomForest" were used to develop the random forest model.

LASSO regression

We used L1-penalized least absolute shrinkage and selection regression (LASSO) for multivariable analyses, augmented with 10-fold cross validation for internal validation [56]. This is a logistic regression model that penalizes the absolute size of the coefficients, where the sum of absolute values of coefficients is multiplied by a weight coefficient λ and then added to the traditional loss function. With larger penalties, the estimates of weaker factors shrink toward zero, so that only the strongest predictors remain in the model. The optimal λ was chosen via 10-fold cross

validation to minimize the average misclassification error. Subsequently, variables identified by LASSO regression analysis were entered into traditional logistic regression models without penalty (as there is no predictor of more interest than others, double selection was not performed). The package “glmnet” in R was used to perform the LASSO regression, and optimal λ was chosen using the cv.glmnet function.

Ensemble Learning

We use bootstrap aggregation (Bagging) to construct a composite learner [36], which connects multiple base learners in parallel to reduce the variance of the ensemble. In this study, we integrated the best BRT, RF, and LASSO regression models after screening, and treated the AUC of the model as a weight to weight the prediction results of the model, and then generate a new model. That is:

New model = $Model_A*[AUC_A/(AUC_A+ AUC_B+ AUC_C)]+ Model_B*[AUC_B/(AUC_A+ AUC_B+ AUC_C)]+ Model_C*[AUC_C/(AUC_A+ AUC_B+ AUC_C)]$

References [36,47-56] were cited in Supplementary methods.

[36] Zhao C, Wu D, Huang J, et al. BoostTree and BoostForest for Ensemble Learning. IEEE transactions on pattern analysis and machine intelligence. 2023 Jul;45(7):8110-8126.

[47] Kraemer MU, Sinka ME, Duda KA, et al. The global distribution of the arbovirus vectors Aedes aegypti and Ae. albopictus. eLife. 2015 Jun 30;4:e08347.

[48] Pandit PS, Doyle MM, Smart KM, et al. Predicting wildlife reservoirs and global vulnerability to zoonotic Flaviviruses. Nature communications. 2018 Dec 21;9(1):5425.

[49] Shearer FM, Longbottom J, Browne AJ, et al. Existing and potential infection

risk zones of yellow fever worldwide: a modelling analysis. The Lancet Global health. 2018 Mar;6(3):e270-e278.

[50] Wang T, Fan ZW, Ji Y, et al. Mapping the Distributions of Mosquitoes and Mosquito-Borne Arboviruses in China. Viruses. 2022 Mar 27;14(4).

[51] Che TL, Jiang BG, Xu Q, et al. Mapping the risk distribution of Borrelia burgdorferi sensu lato in China from 1986 to 2020: a geospatial modelling analysis. Emerging microbes & infections. 2022 Dec;11(1):1215-1226.

[52] Zhao GP, Wang YX, Fan ZW, et al. Mapping ticks and tick-borne pathogens in China. Nature communications. 2021 Feb 17;12(1):1075.

[53] Yao H, Wang Y, Mi X, et al. The scrub typhus in mainland China: spatiotemporal expansion and risk prediction underpinned by complex factors. Emerging microbes & infections. 2019;8(1):909-919.

[54] Walter M, Vogelgesang JR, Rubel F, et al. Tick-Borne Encephalitis Virus and Its European Distribution in Ticks and Endothermic Mammals. Microorganisms. 2020 Jul 17;8(7).

[55] Zhang J, Yue M, Hu Y, et al. Risk prediction of two types of potential snail habitats in Anhui Province of China: Model-based approaches. PLoS neglected tropical diseases. 2020 Apr;14(4):e0008178.

[56] Liang W, Liang H, Ou L, et al. Development and Validation of a Clinical Risk Score to Predict the Occurrence of Critical Illness in Hospitalized Patients With COVID-19. JAMA internal medicine. 2020 Aug 1;180(8):1081-1089.

**Additional tables:**

**Supplementary Table S1: Inclusion criteria for confirmed infections in this study.**

| Species | Methods |
|---|---|
| Vectors | (1) molecular detection with PCR or sequencing; |
| | (2) isolation and cultivation of pathogens from samples; |
| Hosts | (1) molecular detection with PCR or sequencing; |
| | (2) isolation and cultivation of pathogens from samples; |
| Humans | (1) molecular detection with PCR or sequencing; |
| | (2) isolation and cultivation of pathogens from samples; |
| | (3) a four-fold increase in titer or a seroconversion of specific antibodies in blood sera, and must be confirmed by VNT. |

USUV: Usutu virus; PCR, Polymerase Chain Reaction; VNT: virus neutralization tests.

**Supplementary Table S2: Variables extracted from included studies.**

| Variables | Explanation |
|---|---|
| Title | Article title of the included article. |
| Authors | All authors of the included article. |
| Publication year | Publication year of the included article. |
| Study period | The start and end time of the research. |
| Sampling location | Locations of sampling at different level (country, province, county, explicit coordinates). |
| Detection method | Detection methods used for USUV (molecular assay, isolation and cultivation, serological assay). |
| Species | The tested vector/animal species. |
| Total number | Total number of tested samples. |
| Positive number | Positive number of tested samples. |
| Age[a] | The age or age group of each USUV-infected individual. |
| Sex[a] | The sex of each USUV-infected individual. |
| Clinical manifestation[a] | The clinical symptoms of each USUV-infected individual. |
| Virus strain[a] | The identified virus strains of USUV isolated from human cases. |
| Co-infection[a] | Results on laboratory testing for other viruses. |

a: Only in human related research.

**Supplementary Table S3: Factors potentially associated with USUV infections used in the modeling analysis.**

| Variable | Variable properties | Content | Method | Usage | Reference |
|---|---|---|---|---|---|
| Bio1-19 | Ecoclimatic | USUV infections in Europe | Model: Maxent and SEIR model | Used in the ecological niche model of USUV infections. | Cheng Y, et al. [30] |
| | | The occurrence of USUV infections in birds in central Europe | Model: BIOCLIM, DOMAIN Maxent, GLM, RF, and BRT model | Used in the ecological niche model of USUV infections in birds. | Walter M, et al. [57] |
| | | Monkeypox disease in the Congo Basin | Model: Logistic regression and Maxent | Used in the ecological niche model of monkeypox. | Fuller T, et al. [58] |
| | | Monkeypox virus infections in global range | Model: BRT model | Used in the ecological niche model of monkeypox. | Sun YQ, et al. [59] |
| | | Natural focal diseases in global range | Model: Maxent model | Used in the ecological niche model of natural focal diseases. | Magalhães AR, et al. [60] |
| | | SFGR infections in global range | Model: BRT model | Used in the ecological niche model of SFGR infections. | Zhang YY, et al. [61] |
| | | USUV infections in Austria | Model: SEIR model | Used in the ecological niche model of USUV infections. | Brugger K, et al. [62] |
| Land cover | Environmental | The occurrence of USUV infections in birds in central Europe | Model: BIOCLIM, DOMAIN Maxent, GLM, RF, and BRT model | Used in the ecological niche model of USUV infections in birds. | Walter M, et al. [57] |
| | | Emerging zoonotic diseases in global scale | Model: Logistic regression | Used in the ecological niche model of emerging zoonotic diseases. | Allen T, et.al. [63] |
| | | Monkeypox virus infections in global range | Model: BRT model | Used in the ecological niche model of monkeypox. | Sun YQ, et al. [59] |

| | | | | | |
|---|---|---|---|---|---|
| | | Natural focal diseases in global range | Model: Maxent | Used in the ecological niche model of natural focal diseases. | Magalhães AR, et al. [60] |
| | | SFGR infections in global range | Model: BRT model | Used in the ecological niche model of SFGR infections. | Zhang YY, et al. [61] |
| Elevation | Environmental | The occurrence of USUV infections in birds in central Europe | Model: BIOCLIM, DOMAIN Maxent, GLM, RF, and BRT model | Used in the ecological niche model of USUV infections in birds. | Walter M, et al. [57] |
| | | Monkeypox virus infections in global range | Model: BRT model | Used in the ecological niche model of monkeypox. | Sun YQ, et al. [59] |
| | | SFGR infections in global range | Model: BRT model | Used in the ecological niche model of SFGR infections. | Zhang YY, et al. [61] |
| Leaf area index | Environmental | Monkeypox disease in the Congo Basin | Model: Logistic regression and Maxent model | Used in the ecological niche model of monkeypox. | Fuller T, et.al. [58] |
| | | Monkeypox virus infections in global range | Model: BRT model | Used in the ecological niche model of monkeypox. | Sun YQ, et al. [59] |
| | | SFGR in global range | Model: BRT model | Used in the ecological niche model of SFGR infections. | Zhang YY, et al. [61] |
| Population count | Socioeconomic | Monkeypox disease in the Congo Basin | Model: Logistic regression and Maxent model | Used in the ecological niche model of monkeypox. | Fuller T, et.al. [58] |
| | | Emerging zoonotic diseases in global scale | Model: Logistic regression model | Used in the ecological niche model of emerging zoonotic diseases. | Allen T, et.al. [59] |
| | | Monkeypox virus infections in global range | Model: BRT model | Used in the ecological niche model of monkeypox. | Sun YQ, et al. [59] |
| | | Natural focal disease in global range | Model: Maxent model | Used in the ecological niche model of natural focal diseases.. | Magalhães AR, et al. [60] |

| | | | | | |
|---|---|---|---|---|---|
| | | SFGR infections in global range | Model: BRT model | Used in the ecological niche model of SFGR infections. | Zhang YY, et al. [61] |
| Night light index | Socioeconomic | CCHF virus seroprevalence among livestock in Uganda | Model: GLM | Used in the ecological niche model of CCHF virus seroprevalence. | Telford C, et al. [64] |
| Global downscaled GDP | Socioeconomic | Monkeypox virus infections in global range | Model: BRT model | Used in the ecological niche model of monkeypox. | Sun YQ, et al. [59] |
| | | Natural focal diseases in global range | Model: Maxent model | Used in the ecological niche model of natural focal diseases. | Magalhães AR, et al. [60] |
| HDI | Socioeconomic | Natural focal diseases in global range | Model: Maxent | Used in the ecological niche model of natural focal diseases. | Magalhães AR, et al. [60] |
| Gini index | Socioeconomic | Natural focal diseases in global range | Model: Maxent | Used in the ecological niche model of natural focal diseases. | Magalhães AR, et al. [60] |
| Livestock density | Biological | SFGR infections in global range | Model: BRT model | Used in the ecological niche model of SFGR infections. | Zhang YY, et al. [61] |
| Vector | Biological | USUV infections in Europe | Model: SEIR model | Used in the ecological niche model of USUV infections | Cheng Y, et al. [30] |
| | | USUV infections in Austria | Model: SEIR model | Used in the ecological niche model of USUV infections | Brugger K, et al. [62] |
| Host | Biological | USUV infections in Europe | Model: SEIR model | Used in the ecological niche model of USUV infections | Cheng Y, et al. [30] |
| | | USUV infections in Austria | Model: SEIR model | Used in the ecological niche model of USUV infections | Brugger K, et al. [62] |

BRT: Boosted regression trees. CCHF: Crimean-Congo Hemorrhagic Fever. GLM: Generalized linear model. Maxent: Maximum entropy model. RF: Random forests. SEIR: Susceptible-exposed-infected-removed. SFGR: Spotted fever group rickettsiae. USUV: Usutu Virus. GDP: Gross domestic product. HDI: Human

development index.

References [30, 57-64] were cited in Table S3.

[30] Cheng Y, Tjaden NB, Jaeschke A, et al. Evaluating the risk for Usutu virus circulation in Europe: comparison of environmental niche models and epidemiological models. International journal of health geographics. 2018 Oct 12;17(1):35.

[57] Walter M, Brugger K, Rubel F. Usutu virus induced mass mortalities of songbirds in Central Europe: Are habitat models suitable to predict dead birds in unsampled regions? Preventive veterinary medicine. 2018 Nov 1;159:162-170.

[58] Fuller T, Thomassen HA, Mulembakani PM, et al. Using remote sensing to map the risk of human monkeypox virus in the Congo Basin. EcoHealth. 2011 Mar;8(1):14-25.

[59] Sun YQ, Chen JJ, Liu MC, et al. Mapping global zoonotic niche and interregional transmission risk of monkeypox: a retrospective observational study. Globalization and health. 2023 Aug 17;19(1):58.

[60] Magalhães AR, Codeço CT, Svenning JC, et al. Neglected tropical diseases risk correlates with poverty and early ecosystem destruction. Infectious diseases of poverty. 2023 Apr 10;12(1):32.

[61] Zhang YY, Sun YQ, Chen JJ, et al. Mapping the global distribution of spotted fever group rickettsiae: a systematic review with modelling analysis. The Lancet Digital health. 2023 Jan;5(1):e5-e15.

[62] Brugger K, Rubel F. Simulation of climate-change scenarios to explain Usutu-virus dynamics in Austria. Preventive veterinary medicine. 2009 Jan 1;88(1):24-31.

[63] Allen, T., Murray, K. A., Zambrana-Torrelio, et al. (2017). Global hotspots and correlates of emerging zoonotic diseases. Nat Commun.

2017 Oct 24;8(1):1124.

[64] Telford C, Nyakarahuka L, Waller L, et al. Spatial prediction of Crimean Congo hemorrhagic fever virus seroprevalence among livestock in Uganda. One health (Amsterdam, Netherlands). 2023 Dec;17:100576.

**Supplementary Table S4: Description of potential explanatory covariates used in the model.**

| Variable | Variable properties | Description | Spatial resolution | Temporal range | Source of data | Website |
|---|---|---|---|---|---|---|
| Climate | Ecoclimatic | | 0° 2.5′ | 2002–2018 | WorldClim | https://www.worldclim.org/ |
| Bio1 | | Annual mean temperature (°C) | | | | |
| Bio2 | | Mean diurnal range (Mean of monthly (max temp-min temp)) (°C) | | | | |
| Bio3 | | Isothermality (Bio2/ Bio7) (*100) | | | | |
| Bio4 | | Temperature seasonality (standard deviation*100) | | | | |
| Bio5 | | Max temperature of warmest month (°C) | | | | |
| Bio6 | | Min temperature of coldest month (°C) | | | | |
| Bio7 | | Annual range of temperature (Bio5- Bio6) (°C) | | | | |
| Bio8 | | Mean temperature of wettest quarter (°C) | | | | |
| Bio9 | | Mean temperature of driest quarter (°C) | | | | |
| Bio10 | | Mean temperature of warmest quarter (°C) | | | | |
| Bio11 | | Mean temperature of coldest quarter (°C) | | | | |
| Bio12 | | Annual precipitation (mm) | | | | |
| Bio13 | | Precipitation of wettest month (mm) | | | | |
| Bio14 | | Precipitation of driest month (mm) | | | | |
| Bio15 | | Precipitation seasonality (Coefficient of variation) | | | | |
| Bio16 | | Precipitation of wettest quarter (mm) | | | | |
| Bio17 | | Precipitation of driest quarter (mm) | | | | |
| Bio18 | | Precipitation of warmest quarter (mm) | | | | |
| Bio19 | | Precipitation of coldest quarter (mm) | | | | |
| Land cover | Environmental | | 0.3 Km | 2002–2019 | European Space Agency | https://maps.elie.ucl.ac.be/CCI/ |
| Cropland | | Percentage coverage of cropland (%) | | | | |
| Mixed cropland and nature | | Percentage coverage of mixed cropland and nature vegetation (%) | | | | |

| | | | | | |
|---|---|---|---|---|---|
| vegetation | | | | | |
| Forest | Percentage coverage of forest (%) | | | | |
| Shrubland | Percentage coverage of shrubland (%) | | | | |
| Mixed tree, shrub and herbaceous | Percentage coverage of mixed tree, shrub and herbaceous (%) | | | | |
| Grassland | Percentage coverage of grassland (%) | | | | |
| Lichens and mosses | Percentage coverage of lichens and mosses (%) | | | | |
| Sparse vegetation land | Percentage coverage of sparse vegetation land (%) | | | | |
| Vegetation flooded | Percentage coverage of vegetation flooded (%) | | | | |
| Urban construction land | Percentage coverage of urban construction land (%) | | | | |
| Bare areas | Percentage coverage of bare areas (%) | | | | |
| Water body | Percentage coverage of inland water body (%) | | | | |
| Ice and snow | Percentage coverage of ice and snow (%) | | | | |
| LAI | Environmental | 8 km | 2002–2019 | Resource and Environment Science and Data Center | https://www.resdc.cn/ |
| Leaf area index | Area of leaves (m²) over a unit of land (m²) | | | | |
| Ele | Environmental | 1 Km | 2010 | EarthEnv (DEM90) | http://www.earthenv.org/ |
| Elevation | Elevation (Km) | | | | |
| Livestock density | Biological | 1 Km | 2010 and 2015 | Food and Agriculture Organization | http://www.fao.org/livestock-systems/en/ |
| Horse | Density of horse (heads per km²) | | | | |
| Pig | Density of pig (heads per km²) | | | | |

| | | | | | |
|---|---|---|---|---|---|
| Chicken | | Density of chicken (heads per km²) | | | |
| Population | Socioeconomic | 1 Km | 2020 | LandScan | https://www.satpalda.com/product/landscan/ |
| Population density | | Density of human population (person per km$^2$) | | | |
| HDI | Socioeconomic | 0° 5′ | 1990–2015 | Aalto University | https://datadryad.org/stash/dataset/doi:10.5061/dryad.dk1j0 |
| Human development index | | Common indicators used to measure the income gap among residents of a country or region | | | |
| NLI | Socioeconomic | 0° 0′30″ | 1992–2013 | The U.S. Air Force Defense Meteorological Satellite Program | https://eogdata.mines.edu/products/dmsp/#v4 |
| Nighttime lights index | | Nighttime lights index | | | |
| Gini | Socioeconomic | 1° | 2010 | Zenodo | https://zenodo.org/record/4635734 |
| Gini coefficient | | Gini coefficient | | | |
| GDP | Socioeconomic | 0° 15′ | 1990, 2025 | NASA Socioeconomic Data and Applications Center | https://sedac.ciesin.columbia.edu/ |
| Global downscaled GDP | | Global downscaled GDP | | | |
| Vector | Biological | 0° 2.5′ | 2002–2023 | The BRT model conducted in this study. | |
| Mosquito index | | The maximum predicted occurrence probability of three main vectors (*Culex pipiens*, *Aedes albopictus*, and *Culiseta longiareolata*) of each cell. | | | |
| Host | Biological | 0° 2.5′ | 2002–2023 | The BRT model conducted in this study. | |
| Birds index | | The maximum predicted occurrence probability of three main hosts (*Turdus merula, Passer domesticus, and Ardea cinerea*) of each cell. | | | |

BRT, Boosted regression trees. GDP, Gross domestic product.

**Supplementary Table S5: Positive proportion of USUV infections in different mosquito species.**

| Genus | Species | Total pool | Positive pool | Positive proportion |
|---|---|---|---|---|
| **All** | | **64175** | **1028** | **1.60%** |
| *Culex spp.* | | **51167** | **980** | **1.92%** |
| | *Culex pipiens* | 42075 | 959 | 2.28%[a] |
| | *Culex neavei* | 3831 | 13 | 0.34% |
| | *Culex perexigus* | 508 | 2 | 0.39% |
| | *Culex univitattus* | 585 | 1 | 0.17% |
| | *Culex antennatus* | 1405 | 2 | 0.14% |
| | *Culex modestus* | 2763 | 3 | 0.11% |
| | *Culex perfuscus* | - | - | - |
| *Culiseta spp.* | | **260** | **2** | **0.77%** |
| | *Culiseta annulata* | 215 | 1 | 0.47% |
| | *Culiseta longiareolata* | 45 | 1 | 2.22%[a] |
| *Aedes spp.* | | **11759** | **44** | **0.37%** |
| | *Aedes albopictus* | 3187 | 31 | 0.97%[a] |
| | *Aedes caspius* | 6871 | 13 | 0.19% |
| | *Aedes vexans* | 1607 | 0 | 0.00% |
| | *Aedes detritus* | 94 | 0 | 0.00% |
| | *Aedes japonicus* | - | - | - |
| | *Aedes minutus* | - | - | - |
| *Anopheles spp.* | | **989** | **2** | **0.20%** |
| | *Anopheles maculipennis* | 647 | 2 | 0.31% |
| | *Anopheles claviger* | 183 | 0 | 0.00% |
| | *Anopheles plumbeus* | 147 | 0 | 0.00% |
| | *Anopheles hyrcanus* | 12 | 0 | 0.00% |
| *Mansonia spp.* | | **-** | **-** | **-** |
| | *Mansonia aurites* | - | - | - |

Bold indicates the results summarized based on Genus.

Only data with a total pool number greater than or equal to 10 are included in the calculation.

a: Three main vectors.

"-" indicates data is not counted by pool or the total number of pool is less than 10, thus the data is not included in the calculation of positive proportion.

**Supplementary Table S6: The importance of each USUV positive wildlife.**

| Species | Positive proportion (%) | Importance1 | Positive proportion (%) | Importance2 | Weighted Importance |
|---|---|---|---|---|---|
| *Turdus merula* | 39.02 | 3 | 8.63 | 16 | 6.64 |
| *Ardea cinerea* | 7.14 | 16 | 13.16 | 9 | 14.04 |
| *Passer domesticus* | 16.67 | 6 | 3.59 | 37 | 14.68 |
| *Phasianus colchicus* | 5.00 | 22 | 50.00 | 3 | 16.68 |
| *Columba palumbus* | 8.36 | 14 | 6.28 | 24 | 16.80 |
| *Apus apus* | 5.52 | 20 | 11.54 | 11 | 17.48 |
| *Cyanistes caeruleus* | 18.75 | 5 | 0.00 | 56 | 19.28 |
| *Cygnus olor* | 13.33 | 8 | 0.00 | 57 | 21.72 |
| *Parus major* | 5.04 | 21 | 4.65 | 30 | 23.52 |
| *Turdus philomelos* | 7.19 | 15 | 1.04 | 46 | 23.68 |
| *Erithacus rubecula* | 1.85 | 33 | 19.23 | 5 | 25.16 |
| *Accipiter gentilis* | 4.48 | 24 | 5.26 | 28 | 25.12 |
| *Larus argentatus* | 6.52 | 18 | 2.38 | 42 | 24.72 |
| *Strix aluco* | 1.84 | 35 | 15.71 | 7 | 27.16 |
| *Garrulus glandarius* | 1.84 | 34 | 12.50 | 10 | 27.28 |
| *Columba livia* | 3.61 | 28 | 5.60 | 27 | 27.72 |
| *Hirundo rustica* | 4.65 | 23 | 2.78 | 40 | 27.76 |
| *Pica pica* | 1.10 | 38 | 13.53 | 8 | 29.6 |
| *Delichon urbicum* | 6.67 | 17 | 0.00 | 58 | 28.48 |
| *Otus scops* | 6.45 | 19 | 0.00 | 59 | 30.20 |
| *Buteo buteo* | 2.17 | 31 | 4.56 | 32 | 31.28 |
| *Asio otus* | 1.37 | 37 | 7.69 | 19 | 31.96 |
| *Corvus corone* | 0.30 | 42 | 9.41 | 14 | 34.16 |
| *Streptopelia decaocto* | 4.32 | 25 | 0.00 | 60 | 34.8 |
| *Sylvia atricapilla* | 0.00 | 47 | 8.76 | 15 | 38.04 |
| *Sturnus vulgaris* | 2.50 | 30 | 0.00 | 61 | 38.68 |
| *Sylvia communis* | 0.00 | 49 | 8.00 | 18 | 40.32 |
| *Lanius collurio* | 0.00 | 44 | 4.65 | 31 | 40.36 |
| *Falco tinnunculus* | 0.85 | 40 | 2.65 | 41 | 40.28 |
| *Sula dactylatra* | 0.00 | 59 | 33.33 | 4 | 43.60 |
| *Acrocephalus scirpaceus* | 0.00 | 43 | 1.75 | 43 | 43.00 |
| *Dendrocopos major* | 0.00 | 57 | 11.11 | 12 | 44.40 |
| *Larus michahellis* | 1.75 | 36 | 0.00 | 62 | 43.28 |
| *Bubo bubo* | 0.00 | 52 | 4.82 | 29 | 45.56 |
| *Sylvia borin* | 0.00 | 48 | 3.03 | 39 | 45.48 |
| *Carduelis chloris* | 1.03 | 39 | 0.00 | 63 | 45.72 |
| *Anas platyrhynchos* | 0.00 | 53 | 3.62 | 36 | 48.24 |
| *Tyto alba* | 0.34 | 41 | 0.00 | 64 | 47.44 |
| *Picus viridis* | 0.00 | 58 | 6.25 | 25 | 48.76 |

| | | | | | |
|---|---|---|---|---|---|
| *Sula sula* | 0.00 | 60 | 6.61 | 22 | 49.36 |
| *Muscicapa striata* | 0.00 | 45 | 0.00 | 65 | 50.60 |
| *Onychoprion fuacatus* | 0.00 | 54 | 1.37 | 44 | 51.20 |
| *Accipiter nisus* | 0.00 | 50 | 0.00 | 66 | 54.48 |
| *Melanitta nigra* | 100.00 | 1 | - | - | - |
| *Strix nebulosa* | 57.14 | 2 | - | - | - |
| *Pipistrellus pipistrellus* | 20.00 | 4 | - | - | - |
| *Carduelis carduelis* | 14.29 | 7 | - | - | - |
| *Bubo scandiacus* | 12.00 | 9 | - | - | - |
| *Oriolus oriolus* | 10.00 | 10 | - | - | - |
| *Parabuteo unicinctus* | 9.52 | 11 | - | - | - |
| *Surnia ulula* | 9.09 | 12 | - | - | - |
| *Bubulcus ibis* | 9.09 | 13 | - | - | - |
| *Gallinula chloropus* | 4.00 | 26 | - | - | - |
| *Fringilla coelebs* | 3.92 | 27 | - | - | - |
| *Parus caeruleus* | 3.08 | 29 | - | - | - |
| *Athene noctua* | 1.97 | 32 | - | - | - |
| *Serinus canaria* | 0.00 | 46 | - | - | - |
| *Pernis apivorus* | 0.00 | 51 | - | - | - |
| *Phalacrocorax carbo* | 0.00 | 55 | - | - | - |
| *Merops apiaster* | 0.00 | 56 | - | - | - |
| *Rhea americana* | - | - | 100.00 | 1 | - |
| *Strix uralensis* | - | - | 75.00 | 2 | - |
| *Scolopax rusticola* | - | - | 15.79 | 6 | - |
| *Upupa epops* | - | - | 9.52 | 13 | - |
| *Ovis aries* | - | - | 8.62 | 17 | - |
| *Sylvia curruca* | - | - | 7.69 | 20 | - |
| *Alectoris rufa* | - | - | 6.92 | 21 | - |
| *Fulica atra* | - | - | 6.56 | 23 | - |
| *Sus scrofa* | - | - | 6.03 | 26 | - |
| *Acrocephalus agricola* | - | - | 4.00 | 33 | - |
| *Spilopelia senegalensis* | - | - | 3.98 | 34 | - |
| *Ciconia ciconia* | - | - | 3.88 | 35 | - |
| *Anser anser* | - | - | 3.39 | 38 | - |
| *Euplectes afer* | - | - | 1.12 | 45 | - |
| *Gyps fulvus* | - | - | 0.91 | 47 | - |
| *Dama dama* | - | - | 0.77 | 48 | - |
| *Passer montanus* | - | - | 0.73 | 49 | - |
| *Capreolus pygargus* | - | - | 0.26 | 50 | - |
| *Emberiza schoeniclus* | - | - | 0.00 | 51 | - |
| *Ficedula hypoleuca* | - | - | 0.00 | 52 | - |
| *Locustella luscinioides* | - | - | 0.00 | 53 | - |
| *Phoenicurus phoenicurus* | - | - | 0.00 | 54 | - |

| | | | | | |
|---|---|---|---|---|---|
| *Aegypius monachus* | - | - | 0.00 | 55 | - |
| *Chlorophonia occipitalis* | - | - | - | - | - |
| *Coloeus monedula* | - | - | - | - | - |
| *Corvus monedula* | - | - | - | - | - |
| *Cosmopsarus regius* | - | - | - | - | - |
| *Delichon urbica* | - | - | - | - | - |
| *Erythrura prasina* | - | - | - | - | - |
| *Gracula religiosa* | - | - | - | - | - |
| *Leucopsar rothschildi* | - | - | - | - | - |
| *Mandingoa nitidula* | - | - | - | - | - |
| *Motacilla alba* | - | - | - | - | - |
| *Panurus biarmicus* | - | - | - | - | - |
| *Parus ater* | - | - | - | - | - |
| *Phoenicurus ochruros* | - | - | - | - | - |
| *Pyrrhula erythaca* | - | - | - | - | - |
| *Pyrrhula pyrrhula* | - | - | - | - | - |
| *Serinus serinus* | - | - | - | - | - |
| *Sitta europaea* | - | - | - | - | - |
| *Sturnus unicolor* | - | - | - | - | - |
| *Taeniopygia guttata* | - | - | - | - | - |
| *Troglodytes troglodytes* | - | - | - | - | - |
| *Turdus iliacus* | - | - | - | - | - |
| *Turdus pilaris* | - | - | - | - | - |
| *Aquila chrysaetos* | - | - | - | - | - |
| *Aquila clanga* | - | - | - | - | - |
| *Aquila heliaca* | - | - | - | - | - |
| *Aquila nipalensis* | - | - | - | - | - |
| *Buteo rufinus* | - | - | - | - | - |
| *Circus aeruginosus* | - | - | - | - | - |
| *Geranoaetus melanoleucus* | - | - | - | - | - |
| *Gypaetus barbatus* | - | - | - | - | - |
| *Gyps rueppelli* | - | - | - | - | - |
| *Haliaeetus pelagicus* | - | - | - | - | - |
| *Neophron percnopterus* | - | - | - | - | - |
| *Pandion haliaetus* | - | - | - | - | - |
| *Trigonoceps occipitalis* | - | - | - | - | - |
| *Aegolius funereus* | - | - | - | - | - |
| *Asio flammeus* | - | - | - | - | - |
| *Glaucidium passerinum* | - | - | - | - | - |
| *Strix nebulosa lapponica* | - | - | - | - | - |
| *Alopochen aegyptiaca* | - | - | - | - | - |

| | | | | | |
|---|---|---|---|---|---|
| *Anser canagicus* | - | - | - | - | - |
| *Anas hottentota* | - | - | - | - | - |
| *Branta canadensis* | - | - | - | - | - |
| *Branta ruficollis* | - | - | - | - | - |
| *Chloephaga poliocephala* | - | - | - | - | - |
| *Mergus squamatus* | - | - | - | - | - |
| *Tachyeres pteneres* | - | - | - | - | - |
| *Larosterna inca* | - | - | - | - | - |
| *Larus crassirostris* | - | - | - | - | - |
| *Larus ridibundus* | - | - | - | - | - |
| *Philomachus pugnax* | - | - | - | - | - |
| *Ardea purpurea* | - | - | - | - | - |
| *Eudocimus ruber* | - | - | - | - | - |
| *Platalea leucorodia* | - | - | - | - | - |
| *Pavo cristatus* | - | - | - | - | - |
| *Tetrao urogallus* | - | - | - | - | - |
| *Ciconia nigra* | - | - | - | - | - |
| *Leptoptilos crumeriiferus* | - | - | - | - | - |
| *Alcedo atthis* | - | - | - | - | - |
| *Bucorvus abyssinicus* | - | - | - | - | - |
| *Falco peregrinus pelegrinoides* | - | - | - | - | - |
| *Caprimulgus europaeus* | - | - | - | - | - |
| *Dromaius novaehollandiae* | - | - | - | - | - |
| *Otis tarda* | - | - | - | - | - |
| *Phoenicopterus ruber* | - | - | - | - | - |
| *Calyptorhynchus baudinii* | - | - | - | - | - |
| *Spheniscus humboldti* | - | - | - | - | - |
| *Struthio camelus* | - | - | - | - | - |
| *Nanger dama* | - | - | - | - | - |
| *Ovis musimon* | - | - | - | - | - |
| *Taurotragus oryx* | - | - | - | - | - |
| *Ailuropoda melanoleuca* | - | - | - | - | - |
| *Canis lupus signatus* | - | - | - | - | - |
| *Chrysocyon brachyurus* | - | - | - | - | - |
| *Lycaon pictus* | - | - | - | - | - |
| *Panthera leo persica* | - | - | - | - | - |
| *Vespertilio superans* | - | - | - | - | - |
| *Acrocodia indica* | - | - | - | - | - |
| *Ceratotherium simum* | - | - | - | - | - |

| | | | | | |
|---|---|---|---|---|---|
| *Mastomys natalensis* | - | - | - | - | - |
| *Rattus rattus* | - | - | - | - | - |
| *Pan troglodytes* | - | - | - | - | - |
| *Shrew Crocidura spp.* | - | - | - | - | - |

Only data with a total number greater than or equal to 10 are included in the calculation of positive proportion, and the detailed number of positive number and total number are shown in Stable 10.

"-" indicates that at least one of "Importance 1" or "Importance 2" has no valid data, so "Weighted Importance" cannot be calculated.

"Importance 1" was the order of the positive proportion of each species sorted from high to low of molecular tests. "Importance 2" was the order of the positive proportion of each species sorted from high to low of serological tests.

**Supplementary Table S7: The sources of the species occurrence data.**

| Species | Data sources |
|---|---|
| *Culex pipiens* | GBIF |
| | VectorMap |
| | By exhaustive literature search from PubMed, Web of Science, medRvix, and bioRvix, 318 articles were included (Appendix 2). |
| *Aedes albopictus* | GBIF |
| | VectorMap |
| | Kraemer MU, et al. [47] |
| *Culiseta longiareolata* | GBIF |
| | VectorMap |
| *Turdus merula* | GBIF |
| *Passer domesticus* | GBIF |
| *Ardea cinerea* | GBIF |

References [47] were cited in Table S7

[47] Kraemer MU, Sinka ME, Duda KA, et al. The global distribution of the arbovirus vectors Aedes aegypti and Ae. albopictus. eLife. 2015 Jun 30;4:e08347.

**Supplementary Table S8: The detailed information of demographic characteristics and clinical manifestations of USUV infection humans in other countries.**

| | | Netherlands | Hungary | Germany | The Czech Republic | The Republic of South Africa | Central African Republic |
|---|---|---|---|---|---|---|---|
| | | 9 | 6 | 2 | 1 | 1 | 1 |
| Period (n) | Before 2011 | 0 | 0 | 0 | 0 | 0 | 1 |
| | After 2012 | 9 | 6 | 2 | 1 | 1 | 0 |
| | Unknown [a] | 0 | 0 | 0 | 0 | 0 | 0 |
| Sex (n) | Male | 3 | 4 | 1 | 0 | 1 | 0 |
| | Female | 4 | 2 | 1 | 1 | 0 | 0 |
| | Unknown [a] | 2 | 0 | 0 | 0 | 0 | 1 |
| Age group (n) | <40 | 1 | 3 | 1 | 0 | 1 | 1 |
| | 41-70 | 6 | 3 | 0 | 1 | 0 | 0 |
| | >70 | 0 | 0 | 0 | 0 | 0 | 0 |
| | Unknown/Unclear [a] | 2 | 0 | 1 | 0 | 0 | 0 |
| Month (n) | Before June | 0 | 0 | 1 | 0 | 0 | 0 |
| | June-September | 0 | 1 | 1 | 1 | 0 | 0 |
| | After September | 0 | 0 | 0 | 0 | 0 | 0 |
| | Unknown [a] | 9 | 5 | 0 | 0 | 1 | 1 |
| Under immunosuppressive status (n) | Yes | 0 | 0 | 0 | 0 | 0 | 0 |
| | No | 7 | 6 | 2 | 1 | 1 | 1 |
| | Unknown [a] | 2 | 0 | 0 | 0 | 0 | 0 |

(Continued)

| | | | | | | | |
|---|---|---|---|---|---|---|---|
| Virus lineage (n) | Europe 2 | 0 | 1 | 0 | 0 | 0 | 0 |
| | Europe 3 | 2 | 0 | 1 | 0 | 0 | 0 |
| | Europe 4 | 0 | 0 | 0 | 0 | 0 | 0 |
| | Africa 3 | 0 | 0 | 0 | 0 | 0 | 0 |
| | Untyped [a] | 7 | 5 | 1 | 1 | 1 | 1 |
| Co-infection (n) | Yes | 6 | 0 | 1 | 0 | 0 | 0 |
| | WNV infection | 6 | 0 | 1 | 0 | 0 | 0 |
| | TOSV infection | 0 | 0 | 0 | 0 | 0 | 0 |
| | No | 1 | 5 | 1 | 0 | 1 | 0 |
| | Undetected [a] | 2 | 1 | 0 | 1 | 0 | 1 |
| Symptoms (n) | Asymptomatic | 9 | 5 | 2 | 0 | 0 | 0 |
| | Encephalitis/Meningitis | 0 | 1 | 0 | 1 | 0 | 0 |
| | Fever | 0 | 0-1 | 0 | 1 | 0 | 1 |
| | Headache | 0 | 0-1 | 0 | 1 | 0 | 0 |
| | Rash | 0 | 0-1 | 0 | 0 | 0 | 1 |
| | Asthenia | 0 | 0-1 | 0 | 0 | 0 | 0 |
| | Myalgia | 0 | 0-1 | 0 | 0 | 0 | 0 |
| | Unknown [a] | 0 | 0 | 0 | 0 | 1 | 0 |

a: Cases with incomplete information.

WNV: West Nile virus. TOSV: Toscana virus.

**Supplementary Table S9: Positive proportion of USUV in different livestock.**

| | Total number | Positive number | Positive proportion (%) |
|---|---|---|---|
| **All** | **16620** | **1417** | **8.53%** |
| Horse | 13772 | 1310 | 9.51% |
| Chicken | 634 | 49 | 7.73% |
| Pig | 43 | 2 | 4.65% |
| Dog | 1369 | 44 | 3.21% |
| Donkey | 322 | 10 | 3.11% |
| Sheep | 480 | 2 | 0.42% |
| Turkey | - | - | - |
| Goose | - | - | - |
| Duck | - | - | - |

"-" indicates the total number is less than 10, thus the data is not included in the calculation of positive proportion.

**Supplementary Table S10: Positive proportion of USUV in different species of wildlife.**

| Class | Order | Species | Molecular test | | | Serology test | | |
|---|---|---|---|---|---|---|---|---|
| | | | Total number | Positive number | Positive proportion (%) | Total number | Positive number | Positive proportion (%) |
| All | | | 11289 | 1293 | 11.45 | 11712 | 579 | 4.94 |
| Aves | | | 11279 | 1291 | 11.45 | 9193 | 483 | 5.25 |
| | **Passeriformes** | | **7940** | **1143** | **14.40** | **3191** | **168** | **5.26** |
| | | *Acrocephalus scirpaceus* | 12 | 0 | 0.00 | 286 | 5 | 1.75 |
| | | *Acrocephalus agricola* | - | - | - | 25 | 1 | 4.00 |
| | | *Carduelis carduelis* | 14 | 2 | 14.29 | - | - | - |
| | | *Carduelis chloris* | 97 | 1 | 1.03 | 56 | 0 | 0.00 |
| | | *Chlorophonia occipitalis* | - | - | - | - | - | - |
| | | *Coloeus monedula* | - | - | - | - | - | - |
| | | *Corvus corone* | 677 | 2 | 0.30 | 202 | 19 | 9.41 |
| | | *Corvus monedula* | - | - | - | - | - | - |
| | | *Cosmopsarus regius* | - | - | - | - | - | - |
| | | *Cyanistes caeruleus* | 16 | 3 | 18.75 | 16 | 0 | 0.00 |
| | | *Delichon urbica* | - | - | - | - | - | - |
| | | *Delichon urbicum* | 15 | 1 | 6.67 | 232 | 0 | 0.00 |
| | | *Emberiza schoeniclus* | - | - | - | 32 | 0 | 0.00 |
| | | *Erithacus rubecula* | 108 | 2 | 1.85 | 26 | 5 | 19.23 |
| | | *Erythrura prasina* | - | - | - | - | - | - |
| | | *Euplectes afer* | - | - | - | 89 | 1 | 1.12 |
| | | *Ficedula hypoleuca* | - | - | - | 12 | 0 | 0.00 |
| | | *Fringilla coelebs* | 51 | 2 | 3.92 | - | - | - |
| | | *Garrulus glandarius* | 380 | 7 | 1.84 | 40 | 5 | 12.50 |
| | | *Gracula religiosa* | - | - | - | - | - | - |
| | | *Hirundo rustica* | 43 | 2 | 4.65 | 36 | 1 | 2.78 |
| | | *Lanius collurio* | 5 | 0 | 0 | 43 | 2 | 4.65 |
| | | *Leucopsar rothschildi* | - | - | - | - | - | - |
| | | *Locustella luscinioides* | - | - | - | 12 | 0 | 0.00 |
| | | *Mandingoa nitidula* | - | - | - | - | - | - |
| | | *Motacilla alba* | - | - | - | - | - | - |
| | | *Muscicapa striata* | 16 | 0 | 0.00 | 22 | 0 | 0.00 |
| | | *Panurus biarmicus* | - | - | - | - | - | - |
| | | *Parus ater* | - | - | - | - | - | - |
| | | *Parus caeruleus* | 195 | 6 | 3.08 | - | - | - |
| | | *Parus major* | 139 | 7 | 5.04 | 43 | 2 | 4.65 |
| | | *Passer domesticus* | 234 | 39 | 16.67 | 502 | 18 | 3.59 |
| | | *Passer montanus* | - | - | - | 137 | 1 | 0.73 |
| | | *Phoenicurus ochruros* | - | - | - | - | - | - |

| | | | | | | |
|---|---|---|---|---|---|---|
| *Phoenicurus phoenicurus* | - | - | - | 32 | 0 | 0.00 |
| *Pica pica* | 2642 | 29 | 1.10 | 303 | 41 | 13.53 |
| *Pyrrhula erythaca* | - | - | - | - | - | - |
| *Pyrrhula pyrrhula* | - | - | - | - | - | - |
| *Serinus canaria* | 12 | 0 | 0.00 | - | - | - |
| *Serinus serinus* | - | - | - | - | - | - |
| *Sitta europaea* | - | - | - | - | - | - |
| *Sturnus unicolor* | - | - | - | - | - | - |
| *Sturnus vulgaris* | 440 | 11 | 2.50 | 76 | 0 | 0.00 |
| *Sylvia atricapilla* | 41 | 0 | 0.00 | 137 | 12 | 8.76 |
| *Sylvia borin* | 27 | 0 | 0.00 | 33 | 1 | 3.03 |
| *Sylvia communis* | 14 | 0 | 0.00 | 25 | 2 | 8.00 |
| *Sylvia curruca* | - | - | - | 13 | 1 | 7.69 |
| *Taeniopygia guttata* | - | - | - | - | - | - |
| *Troglodytes troglodytes* | - | - | - | - | - | - |
| *Turdus iliacus* | - | - | - | - | - | - |
| *Turdus merula* | 2609 | 1018 | 39.02 | 568 | 49 | 8.63 |
| *Turdus philomelos* | 153 | 11 | 7.19 | 193 | 2 | 1.04 |
| *Turdus pilaris* | - | - | - | - | - | - |
| **Accipitriformes** | **340** | **8** | **2.35** | **878** | **31** | **3.53** |
| *Accipiter gentilis* | 67 | 3 | 4.48 | 76 | 4 | 5.26 |
| *Accipiter nisus* | 103 | 0 | 0.00 | 103 | 0 | 0.00 |
| *Aegypius monachus* | - | - | - | 19 | 0 | 0.00 |
| *Aquila chrysaetos* | - | - | - | - | - | - |
| *Aquila clanga* | - | - | - | - | - | - |
| *Aquila heliaca* | - | - | - | - | - | - |
| *Aquila nipalensis* | - | - | - | - | - | - |
| *Buteo buteo* | 138 | 3 | 2.17 | 570 | 26 | 4.56 |
| *Buteo rufinus* | - | - | - | - | - | - |
| *Circus aeruginosus* | - | - | - | - | - | - |
| *Geranoaetus melanoleucus* | - | - | - | - | - | - |
| *Gypaetus barbatus* | - | - | - | - | - | - |
| *Gyps fulvus* | - | - | - | 110 | 1 | 0.91 |
| *Gyps rueppelli* | - | - | - | - | - | - |
| *Haliaeetus pelagicus* | - | - | - | - | - | - |
| *Neophron percnopterus* | - | - | - | - | - | - |
| *Pandion haliaetus* | - | - | - | - | - | - |
| *Parabuteo unicinctus* | 21 | 2 | 9.52 | - | - | - |
| *Pernis apivorus* | 11 | 0 | 0.00 | - | - | - |
| *Trigonoceps occipitalis* | - | - | - | - | - | - |
| **Strigiformes** | **793** | **30** | **3.78** | **305** | **38** | **12.46** |
| *Aegolius funereus* | - | - | - | - | - | - |

| | | | | | | |
|---|---|---|---|---|---|---|
| *Asio flammeus* | - | - | - | - | - | - |
| *Asio otus* | 73 | 1 | 1.37 | 65 | 5 | 7.69 |
| *Athene noctua* | 152 | 3 | 1.97 | - | - | - |
| *Bubo bubo* | 19 | 0 | 0.00 | 83 | 4 | 4.82 |
| *Bubo scandiacus* | 25 | 3 | 12.00 | - | - | - |
| *Glaucidium passerinum* | - | - | - | - | - | - |
| *Otus scops* | 31 | 2 | 6.45 | 18 | 0 | 0.00 |
| *Strix aluco* | 163 | 3 | 1.84 | 70 | 11 | 15.71 |
| *Strix nebulosa* | 28 | 16 | 57.14 | - | - | - |
| *Strix nebulosa lapponica* | - | - | - | - | - | - |
| *Strix uralensis* | - | - | - | 24 | 18 | 75.00 |
| *Surnia ulula* | 11 | 1 | 9.09 | - | - | - |
| *Tyto alba* | 291 | 1 | 0.34 | 45 | 0 | 0.00 |
| **Anseriformes** | **84** | **36** | **42.86** | **1343** | **46** | **3.43** |
| *Alopochen aegyptiaca* | - | - | - | - | - | - |
| *Anser anser* | - | - | - | 855 | 29 | 3.39 |
| *Anser canagicus* | - | - | - | - | - | - |
| *Anas hottentota* | - | - | - | - | - | - |
| *Anas platyrhynchos* | 35 | 0 | 0.00 | 470 | 17 | 3.62 |
| *Branta canadensis* | - | - | - | - | - | - |
| *Branta ruficollis* | - | - | - | - | - | - |
| *Chloephaga poliocephala* | - | - | - | - | - | - |
| *Cygnus olor* | 15 | 2 | 13.33 | 18 | 0 | 0.00 |
| *Melanitta nigra* | 34 | 34 | 100.00 | - | - | - |
| *Mergus squamatus* | - | - | - | - | - | - |
| *Tachyeres pteneres* | - | - | - | - | - | - |
| **Charadriiformes** | **263** | **5** | **1.90** | **644** | **11** | **1.71** |
| *Larosterna inca* | - | - | - | - | - | - |
| *Larus argentatus* | 46 | 3 | 6.52 | 42 | 1 | 2.38 |
| *Larus crassirostris* | - | - | - | - | - | - |
| *Larus michahellis* | 57 | 1 | 1.75 | 271 | 0 | 0.00 |
| *Larus ridibundus* | - | - | - | - | - | - |
| *Onychoprion fuacatus* | 150 | 0 | 0.00 | 293 | 4 | 1.37 |
| *Oriolus oriolus* | 10 | 1 | 10.00 | - | - | - |
| *Philomachus pugnax* | - | - | - | - | - | - |
| *Scolopax rusticola* | - | - | - | 38 | 6 | 15.79 |
| **Pelecaniformes** | **71** | **2** | **2.82** | **38** | **5** | **13.16** |
| *Ardea cinerea* | 14 | 1 | 7.14 | 38 | 5 | 13.16 |
| *Ardea purpurea* | - | - | - | - | - | - |
| *Bubulcus ibis* | 11 | 1 | 9.09 | - | - | - |
| *Eudocimus ruber* | - | - | - | - | - | - |
| *Phalacrocorax carbo* | 46 | 0 | 0.00 | - | - | - |
| *Platalea leucorodia* | - | - | - | - | - | - |

| | | | | | | |
|---|---|---|---|---|---|---|
| **Columbiformes** | **945** | **52** | **5.50** | **1584** | **89** | **5.62** |
| *Columba livia* | 332 | 12 | 3.61 | 625 | 35 | 5.60 |
| *Columba palumbus* | 335 | 28 | 8.36 | 716 | 45 | 6.28 |
| *Spilopelia senegalensis* | - | - | - | 226 | 9 | 3.98 |
| *Streptopelia decaocto* | 278 | 12 | 4.32 | 17 | 0 | 0.00 |
| **Galliformes** | **20** | **1** | **5.00** | **173** | **18** | **10.40** |
| *Alectoris rufa* | - | - | - | 159 | 11 | 6.92 |
| *Pavo cristatus* | - | - | - | - | - | - |
| *Phasianus colchicus* | 20 | 1 | 5.00 | 14 | 7 | 50.00 |
| *Tetrao urogallus* | - | - | - | - | - | - |
| **Ciconiiformes** | **-** | **-** | **-** | **103** | **4** | **3.88** |
| *Ciconia ciconia* | - | - | - | 103 | 4 | 3.88 |
| *Ciconia nigra* | - | - | - | - | - | - |
| *Leptoptilos crumeriiferus* | - | - | - | - | - | - |
| **Coraciiformes** | **12** | **0** | **0.00** | **-** | **-** | **-** |
| Alcedo atthis | - | - | - | - | - | - |
| Merops apiaster | 12 | 0 | 0.00 | - | - | - |
| **Bucerotiformes** | **-** | **-** | **-** | **-** | **-** | **-** |
| *Bucorvus abyssinicus* | - | - | - | - | - | - |
| *Upupa epops* | - | - | - | 21 | 2 | 9.52 |
| **Falconiformes** | **353** | **3** | **0.85** | **377** | **10** | **2.65** |
| *Falco peregrinus pelegrinoides* | - | - | - | - | - | - |
| *Falco tinnunculus* | 353 | 3 | 0.85 | 377 | 10 | 2.65 |
| **Gruiformes** | **25** | **1** | **4.00** | **259** | **17** | **6.56** |
| *Fulica atra* | - | - | - | 259 | 17 | 6.56 |
| *Gallinula chloropus* | 25 | 1 | 4.00 | - | - | - |
| **Piciformes** | **76** | **0** | **0.00** | **70** | **7** | **10.00** |
| *Dendrocopos major* | 14 | 0 | 0.00 | 54 | 6 | 11.11 |
| *Picus viridis* | 62 | 0 | 0.00 | 16 | 1 | 6.25 |
| **Suliformes** | **176** | **0** | **0.00** | **166** | **23** | **13.86** |
| *Sula dactylatra* | 52 | 0 | 0.00 | 45 | 15 | 33.33 |
| *Sula sula* | 124 | 0 | 0.00 | 121 | 8 | 6.61 |
| **Apodiformes** | **181** | **10** | **5.52** | **52** | **6** | **11.54** |
| *Apus apus* | 181 | 10 | 5.52 | 52 | 6 | 11.54 |
| **Caprimulgiformes** | **-** | **-** | **-** | **-** | **-** | **-** |
| *Caprimulgus europaeus* | - | - | - | - | - | - |
| **Casuariiformes** | **-** | **-** | **-** | **-** | **-** | **-** |
| *Dromaius novaehollandiae* | - | - | - | - | - | - |
| **Otidiformes** | **-** | **-** | **-** | **-** | **-** | **-** |
| *Otis tarda* | - | - | - | - | - | - |
| **Phoenicopteriformes** | **-** | **-** | **-** | **-** | **-** | **-** |
| *Phoenicopterus ruber* | - | - | - | - | - | - |

| | | | | | | |
|---|---|---|---|---|---|---|
| **Psittaciformes** | **-** | **-** | **-** | **-** | **-** | **-** |
| *Calyptorhynchus baudinii* | - | - | - | - | - | - |
| **Rheiformes** | **-** | **-** | **-** | **10** | **10** | **100.00** |
| *Rhea americana* | - | - | - | 10 | 10 | 100.00 |
| **Sphenisciformes** | **-** | **-** | **-** | **-** | **-** | **-** |
| *Spheniscus humboldti* | - | - | - | - | - | - |
| **Struthioniformes** | **-** | **-** | **-** | **-** | **-** | **-** |
| *Struthio camelus* | - | - | - | - | - | - |
| **Mammalia** | **10** | **2** | **20.00** | **2519** | **96** | **3.81** |
| **Artiodactyla** | **-** | **-** | **-** | **2519** | **96** | **3.81** |
| *Capreolus pygargus* | - | - | - | 758 | 2 | 0.26 |
| *Dama dama* | - | - | - | 260 | 2 | 0.77 |
| *Nanger dama* | - | - | - | - | - | - |
| *Ovis aries* | - | - | - | 58 | 5 | 8.62 |
| *Ovis musimon* | - | - | - | - | - | - |
| *Sus scrofa* | - | - | - | 1443 | 87 | 6.03 |
| *Taurotragus oryx* | - | - | - | - | - | - |
| **Carnivora** | **-** | **-** | **-** | **-** | **-** | **-** |
| *Ailuropoda melanoleuca* | - | - | - | - | - | - |
| *Canis lupus signatus* | - | - | - | - | - | - |
| *Chrysocyon brachyurus* | - | - | - | - | - | - |
| *Lycaon pictus* | - | - | - | - | - | - |
| *Panthera leo persica* | - | - | - | - | - | - |
| **Chiroptera** | **10** | **2** | **20.00** | **-** | **-** | **-** |
| *Pipistrellus pipistrellus* | 10 | 2 | 20.00 | - | - | - |
| *Vespertilio superans* | - | - | - | - | - | - |
| **Perissodactyla** | **-** | **-** | **-** | **-** | **-** | **-** |
| *Acrocodia indica* | - | - | - | - | - | - |
| *Ceratotherium simum* | - | - | - | - | - | - |
| **Rodentia** | **-** | **-** | **-** | **-** | **-** | **-** |
| *Mastomys natalensis* | - | - | - | - | - | - |
| *Rattus rattus* | - | - | - | - | - | - |
| **Primates** | **-** | **-** | **-** | **-** | **-** | **-** |
| *Pan troglodytes* | - | - | - | - | - | - |
| **Soricomorpha** | **-** | **-** | **-** | **-** | **-** | **-** |
| *Shrew Crocidura spp.* | - | - | - | - | - | - |

"-" indicates the total number is less than 10, thus the data is not included in the calculation of positive proportion.

**Supplementary Table S11: Kappa and F1 score of the BRT models for main vectors with different sampling ratios.**

| Species | | 1:1 | 1:3 | 1:5 |
|---|---|---|---|---|
| *Culex pipiens* | Kappa (95% CI) | **0.822 (0.819-0.825)** | 0.778 (0.776-0.781) | 0.732 (0.729-0.735) |
| | F1 Score (95% CI) | **0.911 (0.268-0.997)** | 0.838 (0.217-0.990) | 0.782 (0.182-0.983) |
| *Aedes albopictus* | Kappa (95% CI) | **0.906 (0.904-0.909)** | 0.873 (0.870-0.875) | 0.823 (0.820-0.825) |
| | F1 Score (95% CI) | **0.953 (0.302-0.999)** | 0.906 (0.265-0.996) | 0.856 (0.229-0.992) |
| *Culiseta longiareolata* | Kappa (95% CI) | **0.850 (0.840-0.860)** | 0.738 (0.728-0.748) | 0.665 (0.656-0.675) |
| | F1 Score (95% CI) | **0.928 (0.281-0.998)** | 0.813 (0.202-0.987) | 0.734 (0.158-0.976) |

Bold indicates the best model.

**Supplementary Table S12: The RC of significant contributors to the spatial distribution of the main vectors and hosts based on BRT models.**

| Variable | *Aedes albopictus* | *Culex pipiens* | *Culiseta longiareolata* | *Ardea cinerea* | *Passer domesticus* | *Turdus merula* |
|---|---|---|---|---|---|---|
| Bio2 | 2.30 (1.68-3.02) | 2.25 (1.75-2.74) | 3.04 (1.23-7.79) | 1.13 (1.00-1.27) | | |
| Bio3 | | | | | | |
| Bio4 | | | 9.09 (5.85-13.32) | **19.28 (18.88-19.75)** | 12.49 (11.81-13.39) | 14.00 (13.73-14.33) |
| Bio6 | | | | | | 3.89 (3.60-4.15) |
| Bio7 | 7.03 (5.83-8.18) | 3.13 (2.57-3.58) | | | | |
| Bio8 | 4.18 (3.88-4.48) | 12.44 (10.77-14.21) | 3.22 (1.48-6.77) | 2.74 (2.46-3.10) | 11.30 (10.50-11.94) | 0.90 (0.77-1.02) |
| Bio9 | 4.72 (3.96-5.60) | 3.32 (2.89-3.88) | 1.99 (1.12-4.02) | | | |
| Bio13 | | 1.83 (1.47-2.34) | | 2.42 (2.16-2.66) | | |
| Bio14 | 0.61 (0.36-1.04) | 1.68 (1.37-2.11) | 1.11 (0.49-2.75) | 11.43 (10.43-12.36) | **35.51 (34.04-37.30)** | |
| Bio15 | 2.16 (1.55-2.62) | 1.71 (1.34-2.15) | 2.09 (0.81-3.50) | 1.86 (1.54-2.19) | 0.67 (0.53-0.79) | **29.02 (27.05-30.67)** |
| Bio18 | 4.57 (3.42-5.58) | 1.23 (0.86-1.64) | 2.23 (0.98-4.07) | 3.83 (3.40-4.34) | | |
| Bio19 | 3.43 (2.53-4.57) | 1.14 (0.78-1.57) | 20.28 (9.30-35.13) | | 11.46 (9.96-12.74) | 2.36 (2.10-2.69) |
| Cropland | 1.06 (0.72-1.53) | 3.13 (2.44-4.05) | 1.18 (0.62-1.70) | 1.94 (1.63-2.30) | 0.95 (0.87-1.00) | |
| Mixed cropland and nature vegetation | | 0.96 (0.65-1.44) | 4.93 (2.41-8.73) | 3.50 (2.81-4.22) | 2.88 (2.49-3.30) | |
| Forest | | | | 1.35 (1.19-1.47) | | 20.39 (19.88-21.03) |
| Shrubland | 1.44 (0.92-1.94) | | 0.80 (0.30-1.73) | 1.22 (0.97-1.47) | 1.88 (1.61-2.12) | 1.46 (1.28-1.65) |
| Mixed tree, shrub and herbaceous | | | | 0.58 (0.49-0.70) | | |
| Grassland | 0.92 (0.46-1.35) | | | 0.85 (0.71-0.98) | 0.74 (0.62-0.85) | |
| Sparse vegetation land | | | | 0.61 (0.53-0.71) | | |
| Vegetation flooded | | | | 0.88 (0.78-0.97) | | 1.79 (1.59-1.95) |
| Urban | **62.26 (60.28-64.23)** | **52.83 (50.91-54.77)** | **34.69 (19.17-51.08)** | 4.86 (4.51-5.20) | 9.78 (9.63-9.91) | 1.31 (1.22-1.37) |

| | | | | | | |
|---|---|---|---|---|---|---|
| Bare areas | | 0.82 (0.61-1.15) | | 17.77 (16.62-18.91) | | |
| Water body | | 0.87 (0.62-1.16) | | 10.42 (10.14-10.72) | 2.35 (2.24-2.44) | 3.71 (3.49-3.94) |
| LAI | | | 2.18 (0.96-4.05) | | | |
| Elevation | 1.09 (0.75-1.56) | 4.89 (4.38-5.79) | 2.53 (1.65-4.61) | 1.66 (1.51-1.82) | 3.42 (3.24-3.67) | 1.65 (1.56-1.77) |
| Horse | | 2.24 (1.70-2.88) | 2.39 (1.20-4.99) | 1.15 (1.00-1.34) | 0.90 (0.73-1.02) | |
| Pig | 4.11 (3.21-4.97) | 1.29 (1.03-1.60) | 1.33 (0.63-2.42) | 9.35 (8.69-10.31) | 3.84 (3.84-4.35) | 14.26 (13.45-15.24) |
| Chicken | | 4.12 (3.36-4.65) | 1.91 (0.81-4.03) | 1.16 (1.51-1.82) | 1.87 (1.65-2.11) | 5.24 (5.01-5.50) |

Bold indicates the variable with the highest RC for each species.

We only demonstrated effects with median RC >0.5%.

RC, relative contribution; LAI, leaf area index; BRT, boosted regression trees

**Supplementary Table S13: Comparison of BRT model performance for USUV niche modelling by using different thresholds for polygon and different sampling ratios.**

| Sampling ratio | Threshold | AUC | Accuracy | Sensitivity | Specificity | F1 Score | Kappa |
|---|---|---|---|---|---|---|---|
| 1:1[a] | Point | 0.984 (0.970–0.996) | 0.942 (0.939, 0.945) | 0.954 (0.950, 0.958) | 0.931 (0.925, 0.935) | 0.943 (0.293, 0.998) | 0.884 (0.878, 0.891) |
| | 400 $Km^2$ | 0.989 (0.977–0.997) | 0.952 (0.949, 0.955) | 0.968 (0.965, 0.971) | 0.936 (0.931, 0.940) | 0.953 (0.301, 0.999) | 0.904 (0.898, 0.909) |
| | 900 $Km^2$ | 0.990 (0.981–0.997) | 0.958 (0.955, 0.961) | 0.955 (0.951, 0.959) | 0.961 (0.958, 0.965) | 0.958 (0.306, 0.999) | 0.916 (0.911, 0.922) |
| 1:3[a] | Point | 0.989 (0.980–0.997) | 0.957 (0.955, 0.959) | 0.958 (0.954, 0.962) | 0.957 (0.954, 0.959) | 0.918 (0.274, 0.997) | 0.890 (0.884, 0.895) |
| | 400 $Km^2$ | 0.990 (0·980–0·997) | 0.955 (0.953, 0.957) | 0.957 (0.954, 0.961) | 0.954 (0.952, 0.956) | 0.914 (0.271, 0.997) | 0.883 (0.878, 0.888) |
| | 900 $Km^2$ | 0.991 (0·984–0·997) | 0.959 (0.957, 0.961) | 0.960 (0.956, 0.963) | 0.959 (0.956, 0.961) | 0.921 (0.276, 0.997) | 0.893 (0.889, 0.898) |
| 1:5[a] | Point | 0.991 (0·981–0·997) | 0.962 (0.960, 0.963) | 0.958 (0.954, 0.962) | 0.963 (0.961, 0.964) | 0.894 (0.256, 0.995) | 0.871 (0.866, 0.876) |
| | 400 $Km^2$ | 0.992 (0·983–0·997) | 0.956 (0.955, 0.958) | 0.964 (0.960, 0.967) | 0.955 (0.953, 0.957) | 0.880 (0.246, 0.994) | 0.854 (0.849, 0.859) |
| | **900 $Km^2$** | **0.992 (0.983–0.998)** | **0.960 (0.958, 0.961)** | **0.959 (0.955, 0.962)** | **0.960 (0.958, 0.961)** | **0.887 (0.251, 0.995)** | **0.862 (0.857, 0.867)** |
| 1:1[b] | Point | 0.934 (0·894–0·966) | 0.876 (0.871, 0.881) | 0.875 (0.868, 0.881) | 0.877 (0.871, 0.884) | 0.876 (0.242, 0.994) | 0.752 (0.743, 0.761) |
| | 400 $Km^2$ | 0.948 (0·924–0·974) | 0.889 (0.885, 0.893) | 0.894 (0.888, 0.899) | 0.884 (0.878, 0.890) | 0.889 (0.252, 0.995) | 0.778 (0.770, 0.786) |
| | 900 $Km^2$ | 0.948 (0·926–0·973) | 0.889 (0.885, 0.893) | 0.887 (0.881, 0.892) | 0.891 (0.885, 0.896) | 0.889 (0.251, 0.995) | 0.777 (0.769, 0.786) |
| 1:3[b] | Point | 0.941 (0·915–0·966) | 0.881 (0.878, 0.884) | 0.863 (0.856, 0.870) | 0.887 (0.883, 0.891) | 0.785 (0.183, 0.984) | 0.704 (0.696, 0.712) |
| | 400 $Km^2$ | 0.952 (0·925–0·975) | 0.895 (0.892, 0.898) | 0.881 (0.875, 0.887) | 0.899 (0.896, 0.903) | 0.808 (0.197, 0.986) | 0.736 (0.729, 0.743) |
| | 900 $Km^2$ | 0.951 (0·929–0·971) | 0.889 (0.886, 0.892) | 0.881 (0.874, 0.886) | 0.892 (0.889, 0.896) | 0.800 (0.192, 0.985) | 0.724 (0.717, 0.731) |
| 1:5[b] | Point | 0.942 (0·913–0·968) | 0.882 (0.880, 0.885) | 0.865 (0.859, 0.872) | 0.886 (0.883, 0.888) | 0.711 (0.144, 0.973) | 0.640 (0.632, 0.648) |
| | 400 $Km^2$ | 0.951 (0·933–0·973) | 0.956(0.955, 0.958) | 0.964(0.960, 0.967) | 0.955(0.953, 0.957) | 0.880 (0.246, 0.994) | 0.854 (0.849, 0.859) |
| | 900 $Km^2$ | 0.952 (0·925–0·972) | 0.899 (0.897, 0.902) | 0.878 (0.872, 0.884) | 0.903 (0.901, 0.906) | 0.743 (0.160, 0.978) | 0.682 (0.675, 0.689) |

a: The “control” grids were randomly sampled from the grids. b: The “control” grids were randomly sampled from the background grids.

Bold indicates the best model.

**Supplementary Table S14: Comparison of RF model performance for USUV niche modelling by using different thresholds for polygon and different sampling ratios.**

| Sampling ratio | Threshold | AUC | Accuracy | Sensitivity | Specificity | F1 Score | Kappa |
|---|---|---|---|---|---|---|---|
| 1:1[a] | Point | 0.985 (0.970–0.997) | 0.942 (0.939, 0.945) | 0.942 (0.937, 0.947) | 0.942 (0.937, 0.946) | 0.941 (0.292, 0.998) | 0.884 (0.877, 0.890) |
| | 400 $Km^2$ | 0.989 (0.979–0.996) | 0.942 (0.939, 0.945) | 0.942 (0.937, 0.947) | 0.942 (0.937, 0.946) | 0.941 (0.292, 0.998) | 0.884 (0.877, 0.890) |
| | 900 $Km^2$ | 0.990 (0.981–0.997) | 0.955 (0.952, 0.958) | 0.962 (0.958, 0.966) | 0.948 (0.944, 0.952) | 0.955 (0.304, 0.999) | 0.910 (0.905, 0.916) |
| 1:3[a] | Point | 0.991 (0.980–0.997) | 0.953 (0.951, 0.955) | 0.970 (0.966, 0.973) | 0.947 (0.945, 0.950) | 0.911 (0.269, 0.997) | 0.880 (0.874, 0.885) |
| | 400 $Km^2$ | 0.991 (0.983–0.997) | 0.953 (0.951, 0.955) | 0.970 (0.966, 0.973) | 0.947 (0.945, 0.950) | 0.911 (0.269, 0.997) | 0.880 (0.874, 0.885) |
| | 900 $Km^2$ | 0.992 (0.984–0.998) | 0.956 (0.954, 0.957) | 0.962(0.958, 0.965) | 0.953(0.951, 0.956) | 0.915(0.271, 0.997) | 0.885(0.880, 0.890) |
| 1:5[a] | Point | 0.992 (0.984–0.997) | 0.954 (0.952, 0.955) | 0.970 (0.966, 0.973) | 0.950 (0.948, 0.952) | 0.874 (0.242, 0.993) | 0.846 (0.840, 0.851) |
| | 400 $Km^2$ | 0.992 (0.983–0.997) | 0.954 (0.952, 0.955) | 0.970 (0.966, 0.973) | 0.950 (0.948, 0.952) | 0.874 (0.242, 0.993) | 0.846 (0.840, 0.851) |
| | **900 $Km^2$** | **0.993 (0.985–0.998)** | **0.958 (0.957, 0.960)** | **0.963(0.960, 0.967)** | **0.958(0.956, 0.959)** | **0.884(0.249, 0.994)** | **0.859(0.854, 0.864)** |
| 1:1[b] | Point | 0.942 (0.912–0.967) | 0.884 (0.879, 0.888) | 0.887 (0.880, 0.893) | 0.881 (0.875, 0.888) | 0.883 (0.247, 0.994) | 0.768 (0.759, 0.777) |
| | 400 $Km^2$ | 0.949 (0.922–0.975) | 0.884 (0.879, 0.888) | 0.887 (0.880, 0.893) | 0.881 (0.875, 0.888) | 0.883 (0.247, 0.994) | 0.768 (0.759, 0.777) |
| | 900 $Km^2$ | 0.947 (0.915–0.967) | 0.890 (0.886, 0.894) | 0.895 (0.889, 0.900) | 0.885 (0.879, 0.891) | 0.890 (0.252, 0.995) | 0.780 (0.772, 0.788) |
| 1:3[b] | Point | 0.946 (0.911–0.969) | 0.881 (0.878, 0.884) | 0.886 (0.879, 0.892) | 0.879 (0.876, 0.883) | 0.788 (0.185, 0.984) | 0.706 (0.699, 0.714) |
| | 400 $Km^2$ | 0.954 (0.930–0.972) | 0.881 (0.878, 0.884) | 0.886 (0.879, 0.892) | 0.879 (0.876, 0.883) | 0.788 (0.185, 0.984) | 0.706 (0.699, 0.714) |
| | 900 $Km^2$ | 0.954 (0.922–0.970) | 0.893 (0.890, 0.896) | 0.865 (0.859, 0.872) | 0.903 (0.899, 0.906) | 0.802 (0.193, 0.986) | 0.730 (0.722, 0.737) |
| 1:5[b] | Point | 0.949 (0.924–0.974) | 0.891 (0.888, 0.894) | 0.871 (0.865, 0.878) | 0.895 (0.892, 0.898) | 0.726 (0.151, 0.975) | 0.660 (0.653, 0.668) |
| | 400 $Km^2$ | 0.955 (0.930–0.970) | 0.891 (0.888, 0.894) | 0.871 (0.865, 0.878) | 0.895 (0.892, 0.898) | 0.726 (0.151, 0.975) | 0.660 (0.653, 0.668) |
| | 900 $Km^2$ | 0.958 (0.939–0.974) | 0.902(0.900, 0.904) | 0.882(0.876, 0.888) | 0.906(0.904, 0.908) | 0.748(0.163, 0.978) | 0.689(0.682, 0.696) |

a: The "control" grids were randomly sampled from the grids. b: The "control" grids were randomly sampled from the background grids.

**Supplementary Table S15: Comparison of LASSO model performance for USUV niche modelling by using different thresholds for polygon and different sampling ratios.**

| Sampling ratio | Threshold | AUC | Accuracy | Sensitivity | Specificity | F1 Score | Kappa |
|---|---|---|---|---|---|---|---|
| 1:1[a] | Point | 0.968 (0.936–0.986) | 0.928 (0.924, 0.931) | 0.950 (0.946, 0.954) | 0.905 (0.899, 0.911) | 0.930 (0.283, 0.998) | 0.856 (0.848, 0.863) |
| | 400 $Km^2$ | 0.980 (0.967–0.995) | 0.948 (0.945, 0.951) | 0.961 (0.958, 0.965) | 0.935 (0.930, 0.939) | 0.949 (0.298, 0.999) | 0.896 (0.890, 0.902) |
| | 900 $Km^2$ | 0.982 (0.968–0.994) | 0.949 (0.946, 0.952) | 0.961 (0.957, 0.964) | 0.938 (0.933, 0.942) | 0.950 (0.299, 0.999) | 0.899 (0.893, 0.904) |
| 1:3[a] | Point | 0.982 (0.965–0.991) | 0.949 (0.947, 0.951) | 0.961 (0.957, 0.964) | 0.945 (0.942, 0.948) | 0.906 (0.265, 0.996) | 0.871 (0.866, 0.877) |
| | 400 $Km^2$ | 0.981 (0.967–0.991) | 0.940 (0.937, 0.942) | 0.963 (0.960, 0.967) | 0.931 (0.929, 0.934) | 0.889 (0.253, 0.995) | 0.848 (0.843, 0.854) |
| | 900 $Km^2$ | 0.983 (0.971–0.994) | 0.946 (0.944, 0.948) | 0.962 (0.959, 0.966) | 0.940 (0.938, 0.943) | 0.900 (0.260, 0.996) | 0.863 (0.858, 0.868) |
| 1:5[a] | Point | 0.982 (0.967–0.994) | 0.951 (0.950, 0.953) | 0.959 (0.955, 0.963) | 0.950 (0.948, 0.952) | 0.869 (0.238, 0.993) | 0.839 (0.834, 0.845) |
| | 400 $Km^2$ | 0.982 (0.969–0.993) | 0.945 (0.943, 0.946) | 0.959 (0.955, 0.962) | 0.942 (0.940, 0.944) | 0.853 (0.228, 0.991) | 0.819 (0.814, 0.825) |
| | **900 $Km^2$** | **0.985 (0.975–0.993)** | **0.955 (0.953, 0.956)** | **0.952 (0.948, 0.956)** | **0.955 (0.954, 0.957)** | **0.876 (0.243, 0.994)** | **0.849 (0.844, 0.854)** |
| 1:1[b] | Point | 0.903 (0.863–0.940) | 0.856 (0.852, 0.861) | 0.879 (0.872, 0.885) | 0.834 (0.827, 0.841) | 0.860 (0.231, 0.992) | 0.713 (0.703, 0.723) |
| | 400 $Km^2$ | 0.893 (0.860–0.928) | 0.841 (0.836, 0.846) | 0.872 (0.865, 0.878) | 0.810 (0.803, 0.818) | 0.845 (0.220, 0.991) | 0.682 (0.672, 0.691) |

| | | | | | | | |
|---|---|---|---|---|---|---|---|
| | 900 $Km^2$ | 0.900 (0.864–0.928) | 0.843 (0.838, 0.848) | 0.861 (0.855, 0.867) | 0.825 (0.818, 0.832) | 0.845 (0.220, 0.991) | 0.686 (0.676, 0.695) |
| 1:3[b] | Point | 0.907 (0.870–0.940) | 0.840 (0.836, 0.843) | 0.876 (0.869, 0.882) | 0.827 (0.823, 0.831) | 0.736 (0.156, 0.977) | 0.625 (0.616, 0.633) |
| | 400 $Km^2$ | 0.907 (0.875–0.936) | 0.842 (0.839, 0.845) | 0.860 (0.854, 0.866) | 0.836 (0.832, 0.840) | 0.733 (0.155, 0.976) | 0.624 (0.617, 0.632) |
| | 900 $Km^2$ | 0.902 (0.868–0.930) | 0.825 (0.822, 0.829) | 0.868 (0.861, 0.874) | 0.811 (0.807, 0.815) | 0.714 (0.145, 0.973) | 0.594 (0.586, 0.601) |
| 1:5[b] | Point | 0.906 (0.875–0.931) | 0.851 (0.848, 0.854) | 0.830 (0.822, 0.837) | 0.855 (0.852, 0.859) | 0.651 (0.116, 0.964) | 0.562 (0.554, 0.570) |
| | 400 $Km^2$ | 0.909 (0.883–0.941) | 0.854 (0.852, 0.857) | 0.852 (0.845, 0.858) | 0.855 (0.852, 0.858) | 0.661 (0.121, 0.965) | 0.574 (0.567, 0.582) |
| | 900 $Km^2$ | 0.904 (0.876–0.931) | 0.839 (0.836, 0.842) | 0.848 (0.842, 0.855) | 0.837 (0.834, 0.840) | 0.638 (0.111, 0.962) | 0.543 (0.536, 0.550) |

a: The "control" grids were randomly sampled from the grids. b: The "control" grids were randomly sampled from the background grids.

**Supplementary Table S16: Model-estimated mean relative contributions of all factors to the spatial distribution of USUV.**

| | BRT | RF | LASSO | Ensemble learning |
|---|---|---|---|---|
| Bio1 | -* | -* | 0.97 (0.65-2.07) | - |
| Bio2 | 1.43 (0.79-2.14) | 3.37 (2.56-3.85) | 0.87 (0.06-2.58) | 1.89 |
| Bio3 | -* | -* | 0.10 (0.01-0.25) | - |
| Bio4 | -* | -* | -** | - |
| Bio5 | -* | -* | 1.11 (0.15-3.31) | - |
| Bio6 | 1.84 (1.19-2.73) | 4.51 (3.67-5.06) | 0.30 (0.03-0.63) | 2.22 |
| Bio7 | 1.29 (0.79-1.76) | 4.41 (3.78-5.05) | -** | 1.92 |
| Bio8 | 2.69 (1.69-3.62) | 3.79 (3.24-4.47) | 0.32 (0.16-0.50) | 2.27 |
| Bio9 | 3.88 (2.13-5.59) | 4.00 (3.31-4.49) | 0.41 (0.10-0.81) | 2.77 |
| Bio10 | -* | -* | 1.86 (0.29-3.58) | - |
| Bio11 | -* | -* | 0.39 (0.12-0.69) | - |
| Bio12 | -* | -* | -** | - |
| Bio13 | 1.38 (0.83-2.15) | 3.56 (2.55-4.09) | -** | 1.67 |
| Bio14 | 1.07 (0.63-1.64) | 3.01 (2.40-3.48) | 0.44 (0.12-0.76) | 1.51 |
| Bio15 | -** | 2.98 (2.12-3.57) | -** | 1.03 |
| Bio16 | -** | 3.47 (2.54-4.14) | -** | 1.19 |
| Bio17 | -* | -* | 0.11 (0.00-0.22) | - |
| Bio18 | 0.98 (0.45-1.75) | 3.27 (2.82-3.73) | -** | 1.44 |
| Bio19 | -** | 2.64 (2.08-3.20) | -** | 0.92 |
| Cropland | -** | 2.15 (1.14-2.73) | 3.29 (1.31-5.19) | 1.83 |
| Mixed cropland and nature vegetation | -** | 2.11 (1.35-2.64) | 1.89 (0.06-6.85) | 1.35 |
| Forest | -** | 2.72 (2.20-3.27) | 0.96 (0.12-2.16) | 1.24 |
| Shrubland | -** | 1.81 (1.01-2.69) | 7.35 (1.58-13.27) | 3.06 |
| Mixed tree, shrub and herbaceous | -** | 1.77 (1.15-2.30) | 2.75 (0.07-9.14) | 1.52 |
| Grassland | -** | 1.12 (0.24-1.94) | 5.02 (1.50-8.71) | 2.06 |
| Lichens and mosses | -* | -* | -** | - |
| Sparse vegetation land | -** | 1.33 (0.58-2.06) | 10.60 (0.32-37.42) | 3.98 |
| Vegetation flooded | -** | 1.68 (0.82-2.40) | 13.94 (5.34-20.84) | 5.20 |
| Urban construction land | 21.11 (8.55-38.65) | 6.43 (5.14-8.28) | 4.00 (0.14-9.80) | 10.53 |
| Bare areas | -** | 1.43 (0.77-2.22) | 6.51 (1.08-22.81) | 2.65 |
| Water body | -** | 1.45 (0.66-2.09) | 1.70 (0.17-4.44) | 1.07 |
| Ice and snow | -* | -* | -** | - |

| | | | | |
|---|---|---|---|---|
| LAI | -** | 2.33 (1.62-2.94) | -** | 0.81 |
| Elevation | -** | 3.04 (2.32-3.70) | -** | 1.05 |
| Horse | 4.66 (3.08-6.81) | 5.03 (4.17-6.07) | -** | 3.25 |
| Pig | 1.04 (0.56-1.70) | 3.66 (3.21-4.11) | -** | 1.59 |
| Chicken | -** | 3.57 (3.02-4.12) | -** | 1.27 |
| Population number | -* | -* | -** | - |
| HDI | 5.12 (3.37-7.13) | 5.88 (4.82-6.69) | 13.48 (4.56-35.95) | 8.15 |
| NLI | -* | -* | -** | - |
| Gini | -** | 1.39 (0.90-1.96) | 1.99 (0.16-5.00) | 1.14 |
| GDP | -* | -* | 0.15 (0.07-0.23) | - |
| **Mosquito index** | **52.36 (34.66-67.35)** | **8.16 (5.95-11.27)** | **15.96 (13.25-20.02)** | **25.51** |
| Birds index | 1.15 (0.58-1.82) | 3.84 (2.64-4.43) | 3.41 (0.23-7.08) | 2.80 |

Bold indicates the most important factor.

*: Excluded due to multicollinearity. **: Values less than 0.05.

## Additional figures:

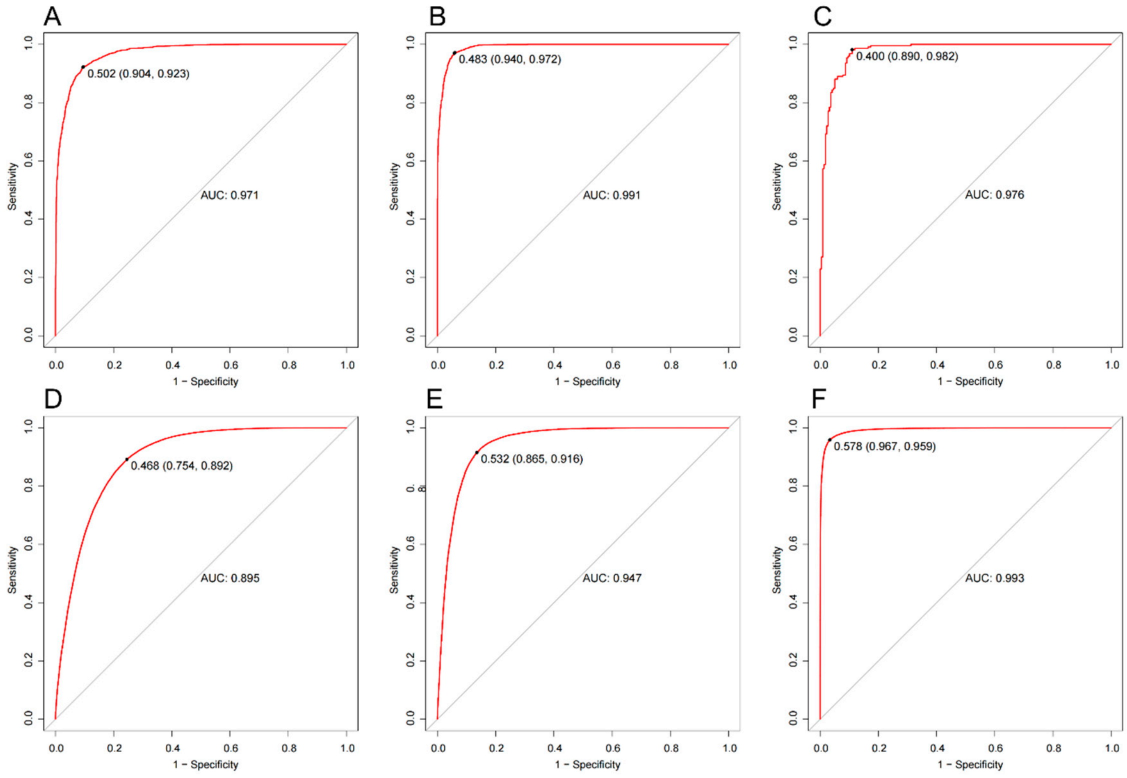


**Figure S1** Predictive performance of the BRT model of the prediction for main vectors and hosts. (A) *Culex pipiens*. (B) *Aedes albopictus*. (C) *Culiseta longiareolata*. (D) *Ardea cinerea*. (E) *Passer domesticus*. (F) *Turdus merula*.

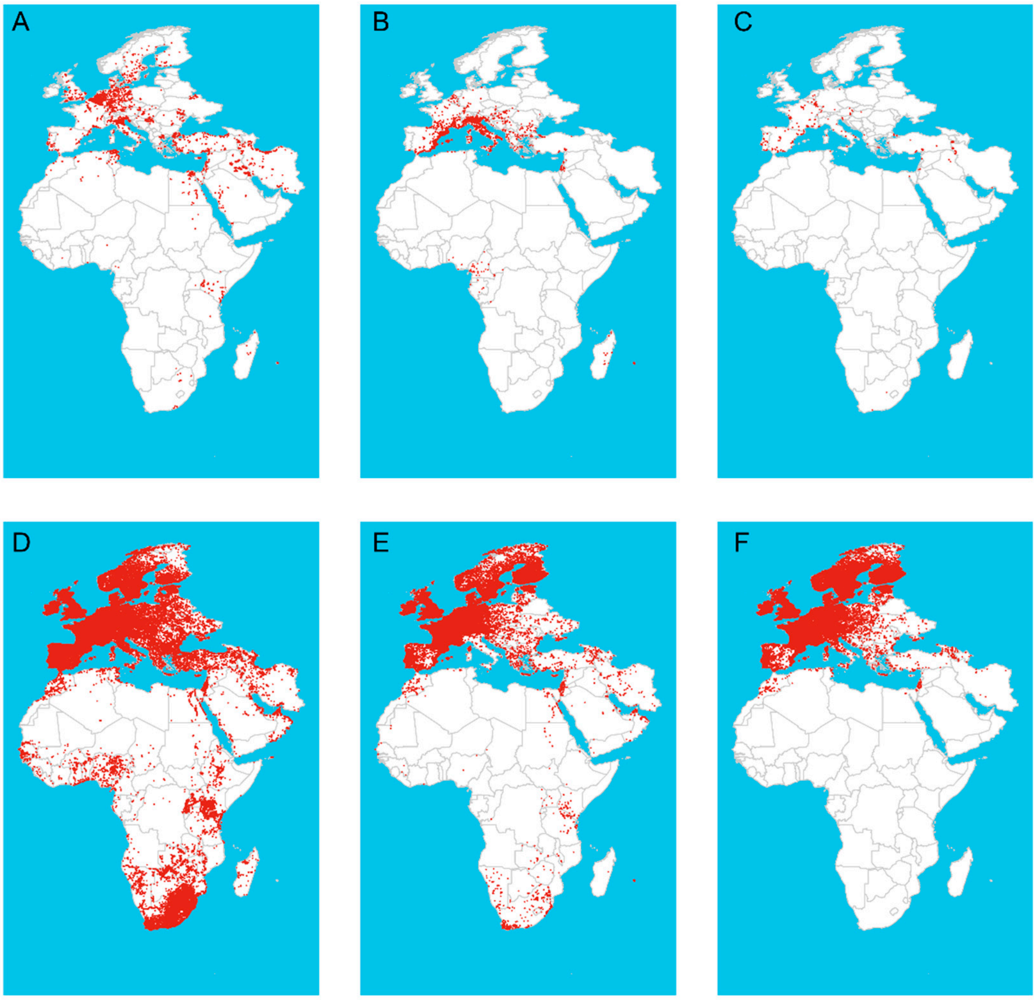


**Figure S2** : The recorded distributions of main vectors and hosts. (A) *Culex pipiens*. (B) *Aedes albopictus*. (C) *Culiseta longiareolata*. (D) *Ardea cinerea*. (E) *Passer domesticus*. (F) *Turdus merula.*

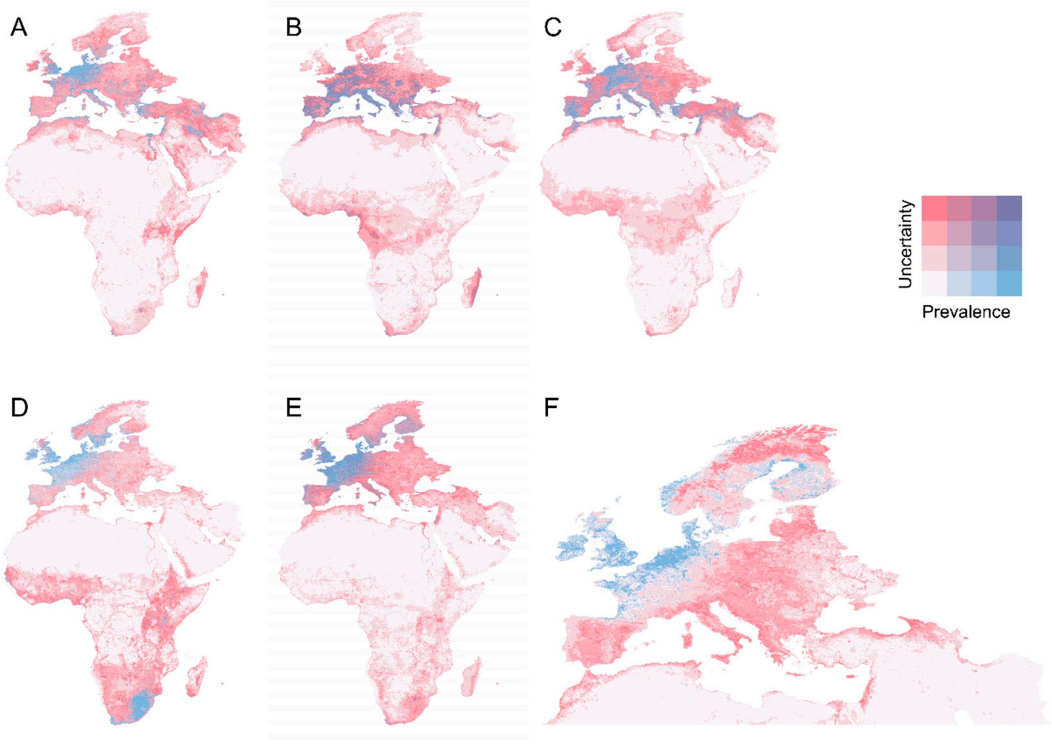


**Figure S3**: Predicted HSI and relative uncertainty of main vectors and hosts, with the colors from light to deep representing the values from low to high.. (A) *Culex pipiens*. (B) *Aedes albopictus*. (C) *Culiseta longiareolata*. (D) *Ardea cinerea*. (E) *Passer domesticus*. (F) *Turdus merula.*
